# Safety of early air travel after treatment of traumatic pneumothorax

**DOI:** 10.3402/ijch.v73.24178

**Published:** 2014-04-10

**Authors:** Frank Sacco, Kelly R. Calero

**Affiliations:** Department of Surgery, Alaska Native Medical Center, Anchorage, AK 99508, USA

Traumatic pneumothorax (PTX) is a commonly encountered injury seen by trauma services and emergency departments on a regular basis. It often occurs in patients who are travelling or vacationing far from home or who have been transferred by air to a higher level of care for treatment for their injuries. The Aerospace Medical Association has presented guidelines stating that PTX is an absolute contraindication to air travel and that air travel is generally safe 2–3 weeks after successful drainage of PTX (1). Current guidelines from the Orlando Regional Medical Center and the British Thoracic Society also recommend delaying air travel for 14 days after radiographic resolution of PTX (2,3). This imposed waiting period can cause significant financial and emotional hardship on patients who have sustained a traumatic PTX and are not permitted to fly home for the recommended 14 days.

The Alaska Native Medical Center (ANMC) in Anchorage, AK, is a 150-bed tertiary referral facility and level II trauma centre. The centre serves 140,000 Alaska native people living in an area twice as large as the state of Texas. ANMC is part of the larger tribal health system that provides primary care at over 200 small villages with facilities ranging from clinics to small regional hospitals staffed by primary care providers. Aeromedical transport is the prime means of moving seriously injured patients to definitive care in Anchorage or Seattle. ANMC is a contained system and is able to follow up patients after discharge to the primary care system.

Many of the patients treated at this facility live in “Bush” Alaska, small, remote villages off the road system accessible only by boat, snowmobile or bush plane. Regional commercial jet service to and from Anchorage with bush plane links to the smaller villages is the primary means of long distance travel throughout the state. Efficient, safe, discharge and follow-up of these patients is very challenging and can be quite expensive. Due to these logistics as well as patients’ desires to get home as soon as possible, ANMC has routinely allowed patients to fly home sooner than the recommended 14-day waiting period. As ANMC is part of a closed health system, it was possible to determine when patients flew and if there were any complications in this group of patients allowed to fly early.

## Methods

Patients admitted to the ANMC between 2001 and 2012 were identified using the hospital's trauma registry (Digital Innovations). A total of 207 patients were identified with a diagnosis of traumatic PTX, hemopneumothorax requiring thoracostomy, or isolated PTX on imaging not treated with chest tube. Only patients who did not live within the local Anchorage area at the time of injury were included. The trauma registry, the electronic health record and charts were used to review clinical data. The travel office within the ANMC assisted in providing the actual dates of flight for patients after discharge from the hospital.

Of the 207 patients initially identified, 127 cases did not meet review criteria. Twenty-one were excluded due to chest tube placement for pleural effusion or empyema without PTX. Thirty-seven were patients requiring long-term care, rehabilitation or follow-up in Anchorage for other injuries. Fifty-two cases were excluded as the patients made their own travel, outside ANMC system; therefore, the day of their flight is unknown. Finally, 17 cases were excluded due to deaths sustained from the primary trauma. Eighty patients met criteria for review.

## Results

**Graph 1:**
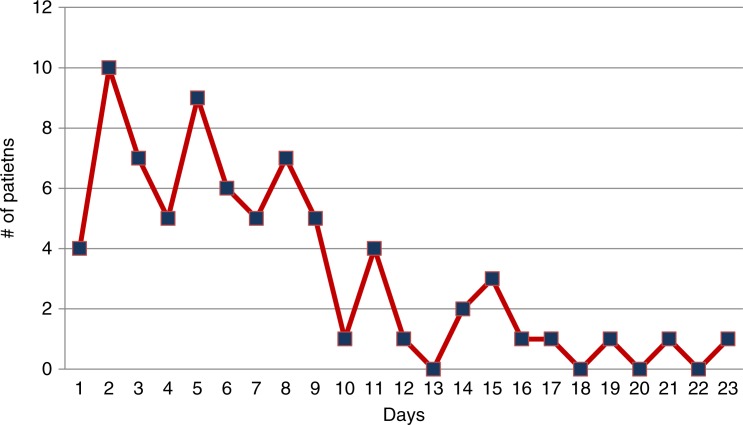
Days between chest tube removal and flight.

**Graph 2:**
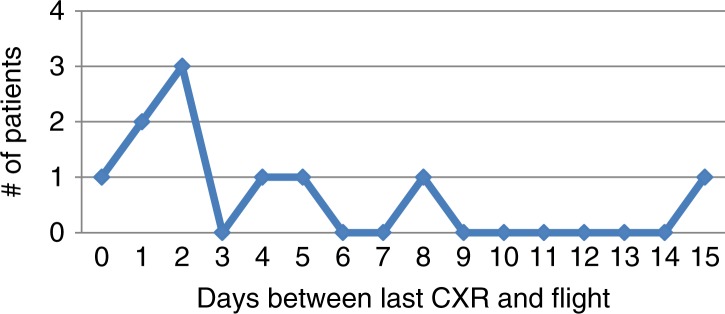
Days between last CXR and flight in patients with pneumothorax.

Ages ranged from 2 to 60 years, with a mean age of 32 years. The group included 63 males and 12 females. Seventy-five cases had a diagnosis of traumatic PTX or hemopneumothorax requiring a chest tube. These patients were followed with chest x-rays and allowed to fly home immediately after discharge from the hospital or were seen in clinic and had follow-up chest x-rays prior to being cleared to return home. The number of days between chest tube removal and flight home was determined (graph 1). The median interval until flight after chest tube removal was 6 days. Fifty-eight patients (77.3%) flew home within 9 days of chest tube removal. No complications were identified in any of these patients either during flight or after returning home. ANMC had 10 patients within the group of 75 patients treated with a chest tube who were cleared to fly with a small stable PTX, none of these patients developed complications. In this sub-group of patients, days between last CXR and flight were tracked (graph 2). Eighty percent of patients flew within 5 days of having a stable residual PTX on CXR. Five patients had isolated PTX on computed tomography (CT) imaging and did not require thoracostomy tube. All 5 flew home after undergoing treatment for other injuries and had no reported complications during flight or after landing.

### Complications

There were no reported complications associated with flight among these 80 patients. There was one re-admission involving a PTX prior to the patient flying. This patient's chest tube had been removed prior to discharge. A post-removal chest x-ray (CXR) was interpreted as subcutaneous emphysema without evidence of PTX. A repeat CXR the next day read the same and he was discharged. He returned to the emergency room on the day of discharge with shortness of breath. A CXR revealed a substantial right side PTX. He was readmitted and the chest tube was reinserted. Upon review, the previous CXRs done after the chest tube removal did reveal an unrecognized expanding PTX; had it been recognized, it would have delayed discharge.

## Discussion

The basis of concern for patients travelling by air with a PTX stems from the properties of gases in enclosed spaces. Boyle's law states that *at constant temperature*, the absolute pressure and the volume of a gas are inversely proportional. Therefore, as atmospheric pressure decreases with an increase in altitude during flight, gas trapped inside a body cavity will expand. This has been estimated to result in a 25–30% increase in volume compared to sea levels in commercial aircraft (2). Expansion of a PTX could potentially lead to respiratory and circulatory compromise.

A small prospective study by Cheatham and Safscak at the Orlando Medical Center looked prospectively at 12 patients flying after resolution of a traumatic PTX. Ten flew after 14 days and had no problems, 2 flew before 14 days and one of the two developed respiratory distress while in flight that was thought consistent with a PTX (2). In contrast, Tam et al. (4) reviewed their experience with 179 patients with PTX after percutaneous lung biopsy that flew within 14 days of the PTX. There were no adverse events in any of these patients and they recommended patients be allowed to fly after sustaining procedure related PTX within 1 day even if there was a small stable residual PTX (4). The Aerospace Medicine Association recommendations currently state that, “Generally, it should be safe to travel by air 2 or 3 weeks after successful drainage of a pneumothorax (or uncomplicated thoracic surgery)” (1). The British Thoracic Society has issued similar guidelines recommending “a minimum delay of one week after full resolution of pneumothorax on chest x-ray, a repeat chest radiograph confirming resolution of pneumothorax before air travel, and 2 week delay following traumatic pneumothorax or uncomplicated thoracic surgery” (3). Unfortunately these recommendations are based on anecdotes, opinion and one small prospective study. Not surprisingly there is fairly wide range of recommendations when surgeons are queried. A survey of thoracic surgeons completed by Szmanski et al. (2010) found wide variability in the practice of thoracic surgeons. Forty-four percent instructed their patients to wait some period of time, an average of 11.7 days, following resolution of PTX prior to air travel (range 0–60 days); however, 45.6% allowed patients to fly with a residual PTX, depending on the size (5). Although ANMC data are retrospective, it does represent a fair sample of patients with accurate follow-up available in a closed healthcare system. Until there is a prospective trial that supports a different approach ANMC will continue to use the following guidelines. Other trauma and thoracic surgeons follow similar strategies (5).

ANMC's current practice for patients treated or identified with traumatic PTX to establish safety for air travel is:

- Obtain a CXR at least 4 hours after chest tube removal; if this shows no PTX or a stable, unchanged small PTX, the patient can be discharged locally or remain in the hospital to await discharge.
- Discharged patients follow up in clinic with a chest x-ray within 48 hours of chest tube removal; if this again shows no PTX or stable unchanged small PTX, the patient is cleared to fly home at that time.
- Patients who remain in the hospital after chest tube removal have a repeat CXR approximately 24 hours later. If this shows no PTX or a stable, unchanged small PTX, they are cleared to fly home at time of hospital discharge.

## Conclusions

Patients with traumatic PTX are discharged every day from trauma centres and hospitals throughout the United States and the world. The implications of delaying clearance for flying have far-reaching economic and social effects. This retrospective review supports the practice of allowing patients with treated traumatic PTX to fly before the widely recommended 14-day waiting period. Current recommendations for air travel following treatment of a traumatic PTX may be too conservative and impose an unnecessary financial and emotional burden on patients and their families. While ANMC's current practice is not based on level I or II evidence, this level of scientific evidence has been difficult to attain for many practical reasons. Retrospective or prospective data from other trauma centres would certainly help validate ANMC recommendations, which ANMC believe are a reasonable approach to this common clinical scenario.
